# Creating a Smartphone App for Caregivers of Children With Atopic Dermatitis With Caregivers, Health Care Professionals, and Digital Health Experts: Participatory Co-Design

**DOI:** 10.2196/16898

**Published:** 2020-10-29

**Authors:** Xiaomeng Xu, Konstadina Griva, Mark Koh, Elaine Lum, Woan Shin Tan, Steven Thng, Josip Car

**Affiliations:** 1 Centre for Population Health Sciences Lee Kong Chian School of Medicine Nanyang Technological University Singapore Singapore; 2 Dermatology Service KK Women's and Children's Hospital Singapore Singapore; 3 Health Services and Outcomes Research Department National Healthcare Group Singapore Singapore; 4 Geriatric Education and Research Institute Singapore Singapore; 5 Skin Research Institute of Singapore Singapore Singapore; 6 Department of Primary Care and Public Health School of Public Health Imperial College London London United Kingdom

**Keywords:** atopic dermatitis, eczema, mobile phone, telehealth

## Abstract

**Background:**

Smartphone apps could support patients and caregivers in disease self-management. However, as patients’ experiences and needs might not always align with clinical judgments, the eliciting and engaging of perspectives of all stakeholders in the smartphone app design process is of paramount importance.

**Objective:**

The aims of this study are to better understand the needs of and challenges facing caregivers and health care professionals (HCPs) who care for children with atopic dermatitis (AD) and to explore the desirable features and content of a smartphone app that would support AD self-management.

**Methods:**

This study adopted a qualitative participatory co-design methodology involving 3 focus group discussions: workshop one focused on caregivers; workshop two engaged with HCPs; and in the last workshop, caregivers and digital health experts were asked to design the wireframe prototype. The participants completed a sociodemographic questionnaire, a technology acceptance questionnaire, and a workshop evaluation form.

**Results:**

Twelve caregivers participated in the first workshop, and 10 HCPs participated in the second workshop. Eight caregivers and 4 digital health experts attended the third workshop. Three superordinate themes that reflected caregivers’ and HCPs’ challenges and needs were identified: *empowerment by education, confusion over treatment*, and *emotional impact*. Workshop participants also raised a series of suggestions on the features and contents of the AD self-management app, which informed the last co-design workshop, and described their needs and challenges. In the last workshop, the participants developed a wireframe prototype of the app following the identified requirements and recommendations.

**Conclusions:**

The co-design approach was found to be a successful way of engaging with the participants, as it allowed them to express their creativity and helped us to articulate the root of the clinical problems. The co-design workshop was successful in creating and generating new ideas and solutions for smartphone app development.

## Introduction

### Background

Atopic dermatitis (AD) is a chronic inflammatory skin condition that affects up to 25% of children worldwide [1,2]. It is characterized by periods of remission and relapse due to variable, often unknown, triggers [3]. Symptoms such as itchiness or soreness can strongly affect children’s daily activities and cause sleep deprivation, which substantially undermine their quality of life (QoL) [4]. The burden of childhood AD extends to their caregivers as well [5,6]. Caregivers’ sleep can be equally affected as parents have to manage their child’s AD symptoms during the night, which can further influence their performance at home and work [7]. In addition, AD, especially childhood AD, requires skilled self-management from caregivers, including coping with a complex regime: caregivers are often required to perform extra duties, such as preparing special meals for their children, engaging in skincare regimens, and cleaning extensively to minimize outbreaks [5,8]. A child with AD can require approximately 2-3 hours of care from their caregivers daily [9]. To prevent relapses, numerous caregivers deem it necessary to cut down on leisure, defer social activities and events, and even sacrifice their jobs to take care of their children [10].

Lack of support leaves caregivers not feeling confident or even failing to manage and provide disease management support for their children’s condition in between visits to the clinic. In clinical practice, it is difficult for health care professionals (HCPs) to educate their patients adequately. Doctors have insufficient time for patient education during the tightly scheduled consultations, and there are limited health educational materials provided in the hospital setting. Caregivers and patients also have difficulty in recalling the health information following a visit to the clinic [11]. The unmet management support needs encourage patients to seek additional information from the internet or friends, and although often most helpful, such apps may also not be trustworthy or easy to use. As such, caregivers must be equipped with supporting tools that are tailored to their needs and help them to properly manage patients’ condition continuously [12].

Digital health tools are increasingly being leveraged to increase patient engagement and facilitate disease self-management for conditions such as diabetes, asthma, dementia, and cancer [13-17]. Smartphone app features can engage patients more in cultivating guidelines-recommended behaviors and providing evidence-based information and advice [18-21]. For example, the disease education feature can give patients insights into their disease condition and enable them to make informed clinical decisions [20,21]; the disease tracking feature can help users detect the possible allergens and help them eliminate aggravating factors and reduce recurrence [20]. It has also been demonstrated to improve clinical outcomes, and 80% of patients consider the service a viable alternative to in-person consultations [22]. Similar to digital management of AD, previous studies comparing AD digital management with traditional in-person care models have demonstrated no significant differences between them regarding clinical outcomes and QoL outcomes [23,24]. In addition, qualitative data showed that doctors found it helpful to use the website in consultations in a pilot randomized control trial [23]. However, when considering the substantial financial savings that digital health interventions in managing AD could reap, digital health could potentially be a promising substitute for in-person AD care [25].

Although many studies document the benefits of digital health tools in terms of supporting users to monitor, treat, and follow-up disease conditions, the quality of apps is a cause for concern, as in many cases, there is a lack of clinical evidence proving the effectiveness of apps. Many of them are not tailored to the user’s needs; this may explain the low adoption and use of apps as part of routine care in clinical practice [26,27]. Working in partnership with stakeholders in the design process can better serve app development. By doing so, developers can more easily identify desirable features for an app as well as obstacles hindering long-term usage; together, app developers and stakeholders could improve the viability, usability, and effectiveness of apps in health care services [28-30].

### Objectives

The co-design approach refers to patients and caregivers working in partnership with staff to improve the viability, usability, and effectiveness of health care services and is considered one of the best ways of improving patients’ experience of services and of guiding developers into understanding the users’ needs [28]. The co-design approach has been used to build self-management apps for chronic diseases such as heart failure [29] and dementia [17,29,31,32]. Co-design has been employed in AD app development before, but relevant web-based information is so sparse that previous experiments cannot easily be repeated by other researchers. Given the fact that self-management is crucial to tackling AD among children, an app involving all stakeholders with detailed procedures is needed to ensure that patients’ needs and voices are sufficiently heard. The objectives of this study are to better understand the needs of and challenges confronting caregivers and HCPs who care for children with AD and to explore the desirable features and content that smartphone apps should offer in supporting AD self-management.

## Methods

### Participant Recruitment

Eligible participants were recruited among those of a baseline cohort in a prior study, who had provided consent to be recontacted for participation in this study [33,34]. Caregivers who met the following inclusion criteria were approached by making phone calls or sending emails: (1) adults providing unpaid care or assistance to children (age<16 years) with AD and (2) prior experience with using a smartphone app to manage health-related issues. The exclusion criteria were as follows: caregivers who did not own a smartphone or had no knowledge of mobile apps. HCPs who have experience in managing AD pediatric patients were recruited directly from the Dermatology Department at the KK Women’s and Children’s Hospital in Singapore. Digital health experts with experience in the field of digital health (app assessment or app development) were recruited face to face from the Lee Kong Chian School of Medicine, Nanyang Technological University.

### Study Design and Settings

Data were collected across three co-design workshops conducted in a specialist hospital in Singapore from September 2018 to March 2019 across two phases (caregivers’ session was held at 7 pm and lasted for 2 hours; HCPs’ session was held at Noon and lasted for 1.5 hours. The design session was held at 7 pm and lasted for 2 hours). The procedure of the co-design workshops is detailed in Textbox 1.

**Co-design workshops.**

**Phase 1**

Content

- Share their problems and difficulties encountered in their daily life
- Propose their needs
- Discuss how the app can be used in their daily life and what features can be adopted by the smartphone app

Caregivers’ session (session duration: 2 hours)

- Caregivers of pediatric patients with atopic dermatitis (n=14)

Health care professionals’ session (session duration: 1.5 hours)

- Pediatric dermatologists (n=8)
- Pharmacists (n=2)

**Phase 2**

Content

- Briefing the need and proposed feature discussed in 4he previous caregivers’ journey sessions
- Top needs were assigned to the participants
- Individual crazy eights exercise to sketch their prototype
- Team sketches
- Revisions
- Present the wireframe to the entire group

Design session (session duration: 2 hours)

- Caregivers (n=6)
- Digital health experts (n=4)

Workshops one and two adopted a focus group discussion format. Workshop one was a focus group discussion with caregivers: 2 researchers (KG and WS) facilitated the group discussion. Additional field notes were collated to capture relevant contextual information (XM and ZL). Semistructured questions were used to guide the focus group discussions. To better serve the research objectives, the semistructured questions used in the previous AD QoL study were designed to address both subjective feelings and objective findings regarding the most disturbing aspects of childhood AD. The topics raised during workshop one are shown in Textbox 2.

Workshop two was a focus group discussion involving HCPs; 2 researchers (JC and KG) facilitated the group discussion. Additional field notes were taken by XX to capture relevant contextual information. Semistructured questions were used to guide focus group discussions. The semistructured questions were designed based on feedback from caregivers in workshop one. They are shown in Textbox 3.

After completion of phase one, a final co-design workshop was conducted (facilitated by JC and XX). As the targeted user of the app is caregivers whose children have AD, the workshop only invited caregivers and digital health experts to design the wireframe prototype. HCPs were not invited in this phase, but we will seek their feedback after the app is developed. After the participants outlined various design requirements in workshops one and two, the facilitator asked participants to design the interface of the app themselves. The app had to address at least one of the top needs mentioned in the previous workshops. The workshop groups discussed and explored the ideas from the drawings and came to an agreement on the top features or ideas they wanted to include in one consolidated master sketch. Groups sketched a single layout that incorporates the top ideas. The teams regrouped and critiqued the wireframe prototype. After the feedback and revision session, the groups discussed and explored the ideas from the drawings and came to an agreement during the development of the consolidated master sketch.

**The topics raised during workshop one.**

Probing questions

- Q1. What were your needs the first 6 months after the symptoms started, from 6 months to a year, or later? Did you feel well informed, especially with respect to (1) disease status, (2) treatment and management guidance, and (3) emotional support?
- Q2. Where do you get the information? Are you satisfied with your knowledge of taking care of your child’s atopic dermatitis (AD)? What is the source of the information?
- Q3. Previous experience in app usage: Has anyone used a smartphone app to manage AD?
- Q4. If there is a smartphone app to help you manage your child’s eczema, please list the top features that you want to have in the app.
- Q5. If there would be a smartphone app with all the information and features that you need, at what frequency do you think you will use it? What are the possible barriers that would keep you from consistent usage?

**The topics raised during workshop two.**

Probing questions

- Q1. How do you educate your patients (their caregivers) during the clinic visit? Are you satisfied with the results?
- Q2. What are caregivers’ need the first 6 months after the symptoms started, from 6 months to a year, or later? Does the education need to change according to (1) different severity, (2) duration of diseases, and (3) child age?
- Q3. Have you considered using smartphone apps to educate your patients (and their caregivers)?
- Q4. What features should an app have to address those problems?
- Q5. Would you mind providing a teleconsultation service to your patients?

### Instruments

The following questionnaires were administered during the co-design workshops: Demographic questionnaire and technology acceptance questionnaire (Multimedia Appendix 1, [35]) and workshop evaluation form (Multimedia Appendix 2).

#### Demographic Questionnaire

Caregiver participants were asked to fill in the demographic questionnaire in workshops 1 and 3, including their date of birth, gender, residential status, highest qualification earned, and main daytime occupation and the child’s date of birth, child’s gender, date of the first diagnosis, and the biggest concern relating to the management of the child’s AD.

#### Technology Acceptance Questionnaire

Caregiver participants were asked to fill in the technology acceptance questionnaire during workshops 1 and 3. The questionnaire was developed by the study team adopting the technology acceptance model [36,37]. The list of questions tested individuals’ knowledge of health care app usage, acceptability, and usability, and it addressed perceived ease of use, perceived usefulness, user satisfaction, and usability [36,37].

#### Workshop Evaluation Form

At the end of each workshop, the participants were asked to fill in the evaluation form and provide feedback. Three questions were asked in the evaluation form: (1) a rating of the workshop, (2) the most helpful aspect of the workshop, and (3) any suggestions for improving the workshops.

### Data Collection and Analysis

Workshops were audio-recorded, transcribed, and managed using NVivo software (QSR International, version 11) [38,39]. The transcript data were first broken down into concepts and reorganized to summarize key issues from each workshop. XX, KG, and JC reviewed and discussed concepts immediately after each workshop so that emerging issues could be explored in subsequent workshops. XM transcribed the audio recordings and processed the observation logs, photos, and written products to contextualize and gain more details about the discussion. Thematic analysis was chosen to analyze the transcriptions from the workshops [22]. An inductive strategy was employed, which allowed for themes to be generated from the data, which were then organized into higher-order themes. The NVivo software program was used to store and code the transcript. First cycle coding was performed on the raw data. Subsequently, researchers (XM and KG) met and compared each other’s codes. An interrater reliability (IRR) test was performed between the researchers’ findings (XM and KG); whenever an acceptable IRR was not achieved, the inconsistencies were jointly discussed until a consensus was reached.

### Ethical Approval

All eligible participants provided informed consent before the workshops. Ethical approval was obtained from the Institutional Review Board of Nanyang Technological University before the commencement of the study (Nanyang Technological University Institutional Review Board: 2018-09-053).

### Public and Patient Involvement

Patients, the public, or caregivers were not directly involved in the development of this study, but they were recruited as participants in this study.

## Results

### Participants’ Characteristics

**Figure 1:**
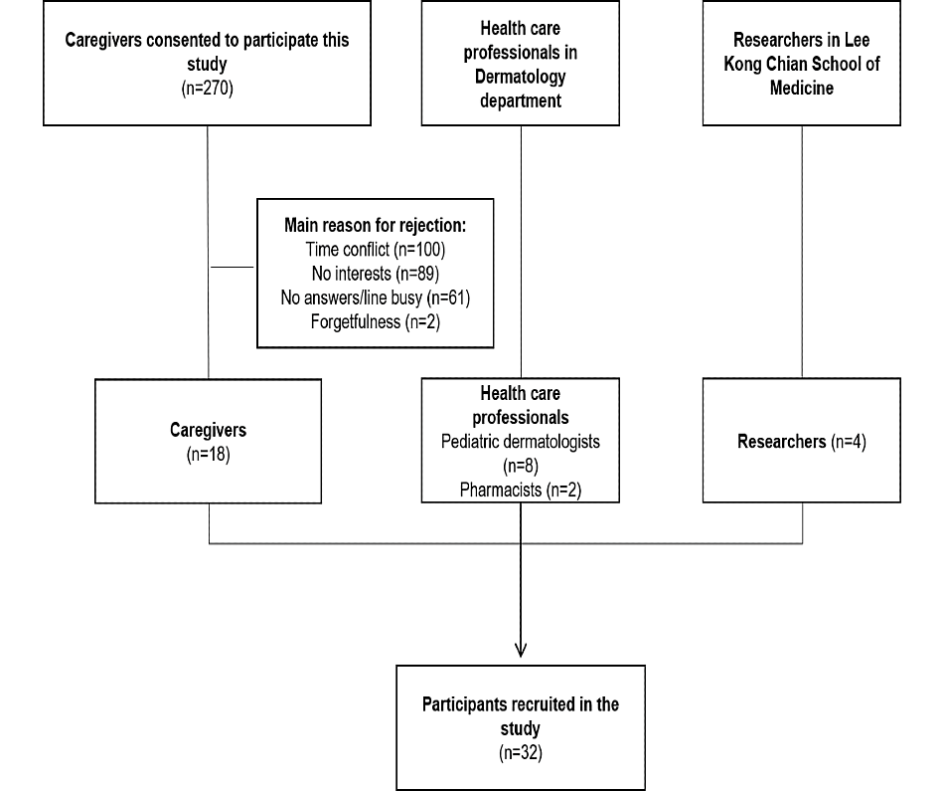
Participants’ recruitment process. Caregivers (n=2) were recruited and participated in 2 workshops (workshop one and workshop three).

The participant recruitment procedure and reasons for exclusion are listed in Figure 1. The characteristics of the caregivers are summarized in Table 1. Out of a total of 270 eligible caregivers, 18 caregivers participated (18/270, 6.7%). The caregivers’ sample included a diverse range of demographics (Table 1): the majority of them were women (14/18, 78%), the mean age of the participants was 37.87 (SD 7.81) years, and the children’s disease duration was 4.72 (SD 4.57) years (range: 1.66-10.83). Pediatric dermatologists (n=8) and pharmacists (n=2) were recruited for the second workshop: their mean age was 35.50 (SD 6.69) years and their mean practice time was 9.76 (SD 6.57) years. Four digital health experts with a mean age of 37.80 (SD 8.12) years attended the third workshop.

The caregivers’ perceptions of using self-management smartphone apps were assessed to test perceived usefulness, ease of use, and user satisfaction (Table 2). A total of 89% (16/18) of the participants found it easy to get mobile tracking on children’s disease, and 89% (16/18) of them felt confident in using a smartphone app to manage children’s AD. A total of 78% (14/18) of the participants believed that using a smartphone app to manage AD would increase the caregiver’s QoL.

**Table 1:** Demographic characteristics of caregiver participants.

Variables and characteristics			Value
**Caregiver**			
	**Gender, n (%)**		
		Female	14 (78)
		Male	4 (22)
	Age (years), mean (SD)		37.87 (7.81)
	**Qualification, n (%)**		
		Bachelor’s degree and above	10 (56)
		Diploma	4 (22)
		O level and below	4 (22)
	Children’s disease duration (years), mean (SD)		4.72 (4.57)
**Health care professional**			
	**Gender, n (%)**		
		Female	8 (80)
		Male	2 (20)
	Age (years), mean (SD)		35.50 (6.69)
	**Specialization, n (%)**		
		Pediatrics dermatology	8 (80)
		Pharmacy	2 (20)
	Practice duration (years), mean (SD)		9.76 (6.57)
**Researcher**			
	**Gender, n (%)**		
		Female	2 (50)
		Male	2 (50)
	Age (years), mean (SD)		37.80 (8.12)
	**Specialization, n (%)**		
		Public health	4 (100)

**Table 2:** Perceived usefulness, ease of use, and user satisfaction of smartphone app among caregivers (strongly agree, agree, neutral, disagree, and strongly disagree).

Perceptions	Strongly agree, number of participants; n (%)	Agree, number of participants; n (%)	Neutral, number of participants; n (%)	Disagree, number of participants; n (%)	Strongly disagree, number of participants; n (%)
Are you aware of the availability of health apps for smartphones	12 (67)	6 (33)	0 (0)	0 (0)	0 (0)
A smartphone self-management app will enable me to get information quickly	5 (28)	10 (56)	3 (17)	0 (0)	0 (0)
A smartphone self-management app will allow my doctor to follow-up the disease from outside of the hospital	4 (24)	10 (56)	4 (24)	0 (0)	0 (0)
A smartphone self-management app will save the time of doctors and nurses	1 (5)	6 (33)	6 (33)	3 (17)	2 (11)
I would find it easy to get mobile tracking on my child’s disease	4 (22)	12 (67)	2 (11)	0 (0)	0 (0)
Using a health app will be clear and understandable	4 (22)	10 (56)	2 (11)	1 (6)	1 (6)
It would be easy for me to become familiar with using a smartphone self-management app	4 (22)	10 (56)	2 (11)	1 (6)	1 (6)
I feel confident in using a smartphone app to manage my child’s AD^a^	2 (11)	14 (78)	2 (11)	0 (0)	0 (0)
I believe using a smartphone app to manage AD will increase my quality of life	3 (17)	11 (61)	4 (22)	0 (0)	0 (0)
I think that I would like to use a smartphone self-management app on a daily basis	3 (17)	12 (67)	3 (17)	0 (0)	0 (0)

^a^AD: atopic dermatitis.

### Contextual Themes

#### Overview

A total of 8 primary themes were identified that reflected caregivers’ and HCPs’ needs and concerns for AD self-management support: (1) understanding of causes, triggers, and symptoms of AD; (2) understanding the disease course and the complications related to AD; (3) which medication to choose and when to use it; (4) generic instruction versus specific instruction; (5) alternative treatments preferable to steroids; (6) guilt and frustration; (7) self-esteem; and (8) the duty to make their child feel better. We subsequently grouped the primary themes into 3 superordinate themes: *empowerment by education*, *confusion over treatment*, and *emotional impact* are foundational issues for support caregivers when helping them to manage a child’s AD. These themes were common to all participant groups and characterized by subthemes to capture the diversity of participant perspectives. Subthemes described by HCPs focused predominantly on steroid usage and understanding of causes and triggers, whereas subthemes described by caregivers additionally touched on their feelings such as guilt, frustration, and self-esteem. These nuanced perspectives on subthemes are further explained below.

#### Empowerment by Education

Both the caregivers and HCPs believed that education enables patients and their caregivers to handle AD and live with AD. The caregiver’s perception of diseases often shapes their attitude about self-management and affects their adherence and capability of engaging in self-management following a medical consultation. Two aspects were highlighted during the discussion: (1) understanding the causes, triggers, and symptoms of AD and (2) understanding disease course and the complications related to AD.

##### Understanding the Causes, Triggers, and Symptoms of AD

The caregiver participants expressed a desire to understand more about AD, especially those caregivers whose children were newly diagnosed with AD. They experienced much confusion during the first few months after the first diagnosis, especially in relation to *cause and triggers* and *symptoms* aspects. They wanted to know what environments or temperatures would prevent them from triggering flare-ups and were concerned as to whether existing symptoms were *normal* (quotes 1 and 2 in Multimedia Appendix 3).

##### Understanding the Disease Course and the Complications Related to AD

Caregivers also described their confusion about the *disease course* and the *complications* related to AD. They claimed that existing support and education received during the consultation might be insufficient and sometimes may not suit their needs. According to one caregiver, “No one told them what happened and what happened next.” Some caregivers did not realize the importance of disease course until they discovered that their child was following another child’s disease pattern (quote 3 in Multimedia Appendix 3.). They also reported that lack of awareness and understanding of the complications related to AD could throw them into uncertainty. Therefore, relevant knowledge should be provided in the first place so as to prepare the caregivers mentally and to help them successfully and smoothly pass the most helpless stage (quote 4 in Multimedia Appendix 3).

HCPs also highlighted the need for a greater understanding of the causes and triggers for patients and their caregivers (quote 5 in Multimedia Appendix 3). However, they also underlined that the caregivers should lower their expectations and accept the truth that their children need to live with the disease. Otherwise, they could develop an irrational attitude, which could hinder them from treating AD properly, particularly if they spend most of the time trying to find a *cure* for AD (quote 6 in Multimedia Appendix 3).

#### Confusion Over Treatment

There was also a consensus that confusion over AD treatment could be another barrier to patients and their caregiver’s proper self-management, one that could have a cascading effect on the outcomes of AD. Caregivers and patients can feel confused over (1) which medication to choose and when to use it, (2) receiving general instructions from a doctor, and (3) treatments that might be preferable to steroids.

##### Which Medication to Choose and When to Use It

Caregivers, especially first-time parents, can feel overwhelmed with all the information available to them when they are selecting medications. There were too many brands for them to choose in the market, and they did not know precisely which medication to choose and when to use it (quote 7 in Multimedia Appendix 3). Confusion in selecting medication was also observed among doctors. Caregivers received different prescriptions from doctors when they switched doctors (quote 8 in Multimedia Appendix 3).

##### Generic Instruction Versus Specific Instruction

Having to administer multiple creams also contributes to the treatment-related burden and confusion experienced by caregivers. Although the administering of lotions itself was not found to be a major issue (as caregivers have usually worked out a routine), they still had pending questions relating to the treatment timing, sequence, and amount, that is, of *applying the creams*. They reported that the doctors always give them guidance in general, but that self-management in daily life is more detailed and difficult. For example, even with the same patient, the severity of AD lesions can vary widely, and so confusion over which cream should be applied to which specific lesion can arise (quote 9 in Multimedia Appendix 3).

##### Alternative Treatments Preferable to Steroids

The option of using less aggressive treatments was discussed during both the caregivers’ session and HCPs’ session. Some parents worried about the side effects of steroids in children and mentioned that they preferred to seek alternative forms of treatment (eg, traditional Chinese medicine and ayurvedic medicine), which are deemed safer (quote 10 in Multimedia Appendix 3.). HCPs, on the other hand, expressed concerns over so-called alternative medications. They claimed that they may contain stronger steroids and that some patients may miss the best treatment opportunities either because they are avoiding or over-applying steroids (quote 11 in Multimedia Appendix 3).

#### Emotional Impact

Peeling and erythema of the skin affect children’s appearance and can be detrimental to their social life. When the skin condition is relapsed, the children’s mood becomes irascible, and their irritable emotions will induce irritation of the skin and worsen their skin condition. Three subthemes relating to the emotional impact of AD were identified during the workshops: (1) guilt and frustration, (2) self-esteem, and (3) the duty to make their child feel better.

##### Guilt and Frustration

The feeling of guilt was repeatedly mentioned by caregivers whenever they talked about their child’s illness. It was difficult for some of the parents to express feelings of guilt, especially the subtler form of guilt, which was expressed in the form of questions (quote 12 in Multimedia Appendix 3). When the parents reflected on their situation, they sometimes regretted things they had done at an earlier stage in life (quote 13 in Multimedia Appendix 3). Guilt may also stem from applying strong medicines to young children. Some felt worried over *putting their kid through something that was not necessary* and the risk that *there is a side effect after the child grows up* (quote 14 in Multimedia Appendix 3). The problem of unhelpful *helpful* advice by others was also raised up repeatedly by the caregivers during the workshop, which could be another cause of feelings of guilt among them (quote 15 in Multimedia Appendix 3). Such advice includes doctors and peers prescribing overuse of steroids as well as unspoken blame and judgment imposed by families, friends, and HCPs.

##### Self-Esteem

Young teenagers are more self-conscious and care more about their body image. When the skin lesions reoccur, children might find it frustrating and sometimes show anger. This is worrying as the emotions may affect the child’s social activities in school and eventually affect their confidence and self-esteem. During the workshop, parents expressed the same worries that when their children got older, AD could affect their children’s self-esteem (quote 16 in Multimedia Appendix 3).

##### The Duty to Make Their Child Feel Better

In addition to experiencing guilt and anger, the parents also expressed more ambiguous concerns that the AD affects their children’s self-esteem. They also felt a strong responsibility to make their children feel better and felt concerned that their children would become more self-aware as they grow older and that AD would affect their emotional health in the long run (quote 17 in Multimedia Appendix 3).

### Ideas and Features of the AD Self-Management App

During the workshops, various functionality requirements were discussed among participants. The most prevalent requirement across the groups was knowledge of the symptoms and on managing the symptoms, medication usage, and triggers. They also expressed a desire to brief caregivers and children about the issues that they might face in the future following diagnosis. This could potentially guide people in transitioning from raising awareness to making concrete actions and plans and developing coping strategies that could help them master everyday challenges.

The HCPs also emphasized that such information should be provided in the right amount in the local language. Excessive information provided during medical consultations may overwhelm both patients and their caregivers. However, inadequate information may also leave them feeling directionless, a feeling which induces them into “going online for relevant information which could lead doctors to spend more consultation time in correcting the wrong online [information].”

The goal is to ensure that users fully understand the requirements and thus reduce confusion over information. To accomplish this, features such as podcasts, chatbots, or web-based lectures were proposed as the potential modes for delivering such information. HCPs also highlighted that the apps should provide other patients’ stories or feedback to debunk the myths concerning or against steroids.

The other requirement discussed during the workshop was the need for patients and caregivers to communicate with HCPs. In this context, participants proposed that technology could be used throughout the whole process of the health care journey, from preparation for the clinical consultation, facilitation of clinical consultation, disease status follow-up, to real-time management after medical consultation. Here, the main goal of the technology would be to help caregivers identify and manage flare-ups; to mitigate confusion; and, at the same time, facilitate HCPs to gain a better understanding of patients’ needs and limitations.

Participants also opined that the technology should enable the user to provide support to others, in this case, by sharing knowledge and experiences with their peers in Singapore. They suggested that apps should be designed to provide social support and facilitate connectedness with others. For example, when other caregivers encounter difficulties when managing flare-ups, their peers could support them and detail their own experiences. Besides, peers can also comfort caregivers emotionally by recognizing their efforts and mitigating their guilt. This might be accomplished using a web-based forum with messaging, chat, or call functions. These were proposed as modes through which other caregivers can provide support for each other.

In addition, both caregivers and HCPs suggested that the app should be personalized. The personalization should embody 2 aspects: (1) features and content should be tailored to each individual’s needs and (2) features and content should suit the local context. In this way, the app could help them with prioritizing and making new choices and with defining their own goals and the small steps necessary for reaching these goals. During the workshops, the participants advised that the app should be personalized to address every patient’s unique circumstances. They recommended that the app should provide all the information needed as soon as a client first uses the app (quote 18 in Multimedia Appendix 3).

Image analysis was also mentioned during the workshop. It was recommended that caregivers could take photographs and use the app to analyze each photograph to measure the severity. They also advised that the app should give advice or suggestions corresponding to the extent and severity of the skin condition, so that clients can get *immediate help without seeing the doctor*.

### Wireframe Prototype Development

The wireframe prototype contained features designed to address the challenges and confusions mentioned in the previous workshops. The *must-have* features and *nice-to-have* features recommended by participants, challenges addressed by the features, and how the final wireframe prototype reflects the features are summarized in Table 3.

**Table 3:** Ideal features for wireframe prototype.

Features		Challenges	Possible solutions
**Must-have features**			
	Knowledge on medication use and their side effects	Which medication to choose and when to use it	Chatbot (Figure 2)
	Knowledge on medication use and their side effects	Generic instruction versus specific instruction	Chatbot (Figure 2)
	Knowledge on medication use and their side effects	Alternative treatments preferable to steroids	Chatbot (Figure 2)
	Knowledge on the symptoms and appropriate management	Understanding the disease course and the complications related to AD^a^	Chatbot (Figure 2)
	Knowledge on the external triggers leading to AD^a^ flares	Understanding the causes, triggers, and symptoms of AD	Chatbot (Figure 2)
	Monitoring of skin conditions	Understanding the causes, triggers, and symptoms of AD	Disease diary (Figure 2)
	Monitoring of skin conditions	Understanding the disease course and the complications related to AD	Journal (Figure 2)
	Communication channels with health care professionals	Understanding the causes, triggers, and symptoms of AD	Teleconsultation (Figure 2)
	Communication channels with health care professionals	Understanding the disease course and the complications related to AD	Teleconsultation (Figure 2)
	Communication channels with health care professionals	Which medication to choose and when to use it	Teleconsultation (Figure 2)
	Communication channels with health care professionals	Generic instruction versus specific instruction	Teleconsultation (Figure 2)
	Communication channels with health care professionals	Alternative treatments preferable to steroids	Teleconsultation (Figure 2)
	Monitoring of external triggers that may lead to AD flares	Understanding the causes, triggers, and symptoms of AD	Disease diary (Figure 2) and disease monitor (Figure 2)
**Nice-to-have features**			
	Debunking the myths about AD	Alternative treatments preferable to steroids	Chatbot (Figure 2)
	Knowledge on the natural history and development of AD	Understanding the causes, triggers, and symptoms of AD	Chatbot (Figure 2)
	Reminders for topical application and medication taking	Which medication to choose and when to use it	Disease monitor (Figure 2)
	Peer support from other caregivers or patients	Understanding the causes, triggers, and symptoms of AD	Forum (Figure 2)
	Peer support from other caregivers or patients	Understanding the disease course and the complications related to AD	Forum (Figure 2)
	Peer support from other caregivers or patients	Which medication to choose and when to use it	Forum (Figure 2)
	Peer support from other caregivers or patients	Generic instruction versus specific instruction	Forum (Figure 2)
	Peer support from other caregivers or patients	Alternative treatments preferable to steroids	Forum (Figure 2)
	Peer support from other caregivers or patients	Guilt and frustration	Forum (Figure 2)
	Peer support from other caregivers or patients	Self-esteem	Forum (Figure 2)
	Peer support from other caregivers or patients	Duty to make their child feel better	Forum (Figure 2)
	Monitoring of caregivers’ and children’s emotions	Guilt and frustration	Disease diary (Figure 2)
	Monitoring of caregivers’ and children’s emotions	Self-esteem	Journal (Figure 2)
	Monitoring of caregivers’ and children’s emotions	Duty to make their child feel better	Journal (Figure 2)
	Emotional support with interactive app features	Guilt and frustration	Forum (Figure 2)
	Emotional support with interactive app features	Self-esteem	Teleconsultation (Figure 2)
	Emotional support with interactive app features	Duty to make their child feel better	Teleconsultation (Figure 2)

^a^AD: atopic dermatitis.

**Figure 2:**
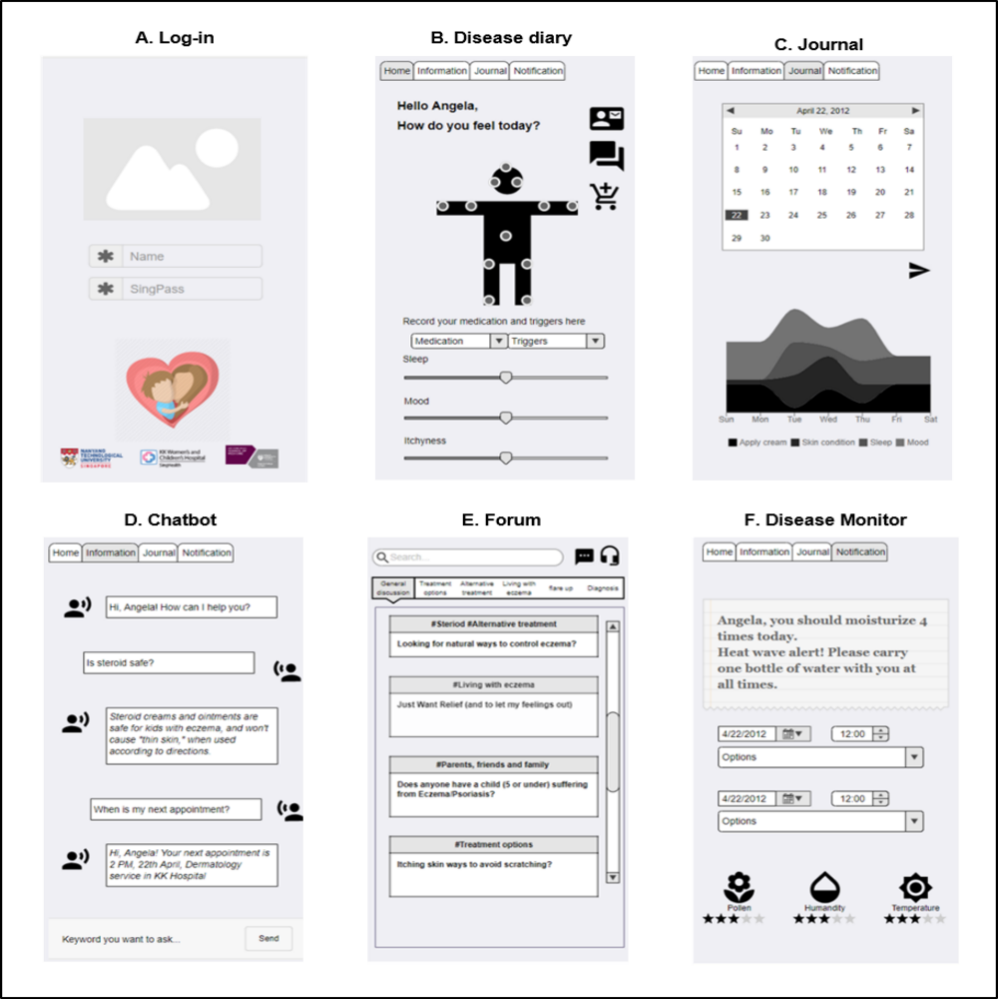
The master sketch of wireframe prototype.

As shown in the master sketch of the prototype, the prototype allows users to log in with their account (Figure 2) and record lesions’ photos of AD in different body parts and provides instructions relating to treatment, symptoms, triggers, and people’s emotions (Figure 2). The *Journal* section enables caregivers to track the patient’s disease condition and could prove useful to their doctor during a visit to the clinic (Figure 2).

Another core feature of the prototype is the education element. Participants designed both the chatbot feature and forum feature, both of which are for educational purposes (Figure 2). The chatbot allows users to access relevant information quickly and easily, which is presented in a clear, understandable format. The forum feature builds a bridge between caregivers and their peers by letting them exchange local information and, to some extent, allowing peers to provide informational and emotional support to users (Figure 2). Participants also designed a notification feature, through which the prototype could remind users to avoid the possible triggers around them and decrease AD relapse (Figure 2).

### Workshop Evaluation Feedback

The workshop evaluation comments indicated that the co-design workshop was successful in creating and generating new ideas and content for smartphone app development. All the participants agreed that the workshop was organized, that its aims were clear, and its activities had been carefully prepared. All the participants felt that the workshop methods used were appropriate for the audience and said that they enjoyed the workshop. However, some issues were also identified in the evaluation feedback. For example, 21% (7/32) of participants highlighted the need to improve workshop time management and felt that the time should be better utilized; they even suggested a longer session.

## Discussion

### Summary

For this study, we organized a series of co-design workshops, which were attended by caregivers living with children with AD, doctors, pharmacists, and digital health experts. During the proceedings, participants jointly explored their needs, preferences, and perceptions in relation to the proposed smartphone self-management app. Phase 1 identified themes that reflected caregivers’ and HCPs’ needs and concerns for AD self-management support, along with suggestions concerning the necessary features and contents of an AD self-management app; the data collected during phase one, therefore, informed phase two, which in turn comprised a smartphone app designing activity. The purpose of the interactive designing exercise was for participants to develop a wireframe prototype. The co-design workshops were designed to encourage social interaction, stimulate creativity, and thus help us to articulate the root of the clinical problems. The findings from the workshops align with those in previous studies, in that co-design interaction can foster a co-creative space and lead to considerable contributions by and involvement of people with chronic disease conditions [40,41].

The wireframe prototype has the potential to solve current AD management challenges. First, information gathered by disease diary and disease monitoring features (Figure 2) in prototype are quite comprehensive with both visual AD symptoms (by taking photos), personal feelings (sleep disturbance, itchiness), psychological impact (mood), and suspected allergens, which could be very helpful for doctors in making precise clinical decisions. In addition, involvement of HCPs in the app development process could ensure that the information provided is reliable. Third, the forum feature (Figure 2) could potentially become a peer support platform. By communicating with each other, caregivers can support each other technically and emotionally and, thus, enable a smooth transition following a consultation and empower caregivers to manage their children’s disease by making them feel competent and capable.

### Research Implications

Some issues were also identified during the workshop. First, it is important to ensure that all the participants fully understand and can keep pace with workshop information and activities—or, to use the colloquialism, *read off the same page*. The participants may interpret questions or tasks differently, and there is a risk of digressing the topic or narrating the same topic repeatedly, which can lead to the workshop being less productive in terms of producing the ideas or thoughts anticipated. Second, at the start of third workshop, we delivered a briefing presentation of the current research and technology used for AD management, to encourage the participants to allow their own needs and challenges to guide app co-design. However, it should be mentioned that the briefing presentation might have limited their thinking, and they may have been inclined to follow the information shared during the briefing session.

### Strengths and Limitations

This study can inform future research studies on co-designing smartphone apps for patients affected by chronic diseases. Participants with a heterogeneous background were invited to co-design the app. This could be considered as a strength of this study as the app design by them will be understandable and beneficial for the wider population regardless of user background. Previous studies have explored treatment needs widely: Batchelor et al [42] tried to identify the uncertainties in eczema treatment that are important to stakeholders. Teasdale et al [43] and Swallow [44] discussed the caregivers’ views of steroid usage [43,44]. However, this chapter goes further in exploring personal experiences, emotional needs, and feelings among both caregivers and HCPs and considering how their points of view could be linked to features of smartphone apps.

However, the scope of the study is limited as the target audience of the app consists entirely of caregivers whose children have AD. However, that limitation alone does not preclude us from being able to satisfy the aims of this study because the content and features caregivers mentioned are to some extent interchangeable. Some of the issues and features caregivers mentioned can also be favored for both children and adults. Second, the co-design process is not rigorously following a participatory co-design framework as continuous evaluation of the prototype was not performed [45]. However, considering that this study has gone through the main process of the framework, this study is still aligned with the overall goal of co-design methodology [29].

Another limitation is that participants were observed to discuss some topics repetitively. Thematic saturation of the workshops was probably less than satisfactory as other themes were barely discussed in phase one, and it might have emerged that we conducted more workshops. However, this limitation can be partly explained as those viewpoints raised repetitively were valued more than other topics from the caregiver’s perspective and, therefore, should be prioritized when designing an AD self-management app. In addition, as the final wireframe prototype is designed by both digital health experts and caregivers, it is difficult for us to differentiate caregivers’ perspectives and digital health experts’ opinions in phase two.

In summary, this study employed a co-design approach to jointly create an AD self-management app wireframe prototype, together with caregivers, HCPs, and digital health experts. The participants’ perceptions and preferences as well as the co-design approach used in this study form a novel contribution to the canon of literature on smartphone app design and the use of digital health tools in an engaging way. Future studies should work on app development as well as invite more stakeholders with separate sessions to test its functionality and usability.

## Appendix

Multimedia Appendix 1 Questionnaire for eczema smartphone app co-design workshop.

Multimedia Appendix 2 Workshop evaluation form.

Multimedia Appendix 3 Illustrative quotes.

## Abbreviations

AD: atopic dermatitis
HCP: health care professional
IRR: interrater reliability
QoL: quality of life
