# Supplementary figures and images for: Creating a Smartphone App for Caregivers of Children With Atopic Dermatitis With Caregivers, Health Care Professionals, and Digital Health Experts: Participatory Co-Design
Source: JMIR Mhealth Uhealth. 2020 Oct 29;8(10):e16898. doi: 10.2196/16898 (PMC7661237; doi:10.2196/16898)

**Appendix 1.** Questionnaire for eczema smartphone app co-design workshop.


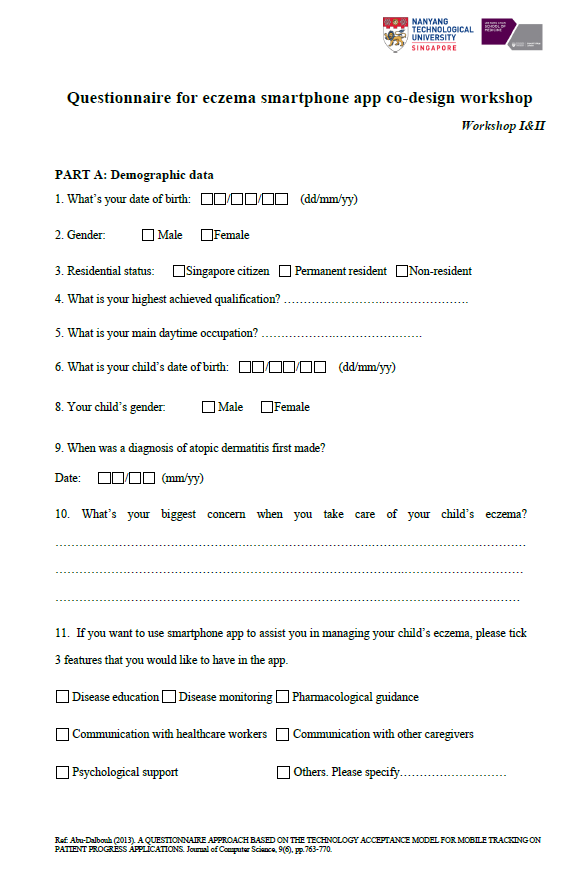


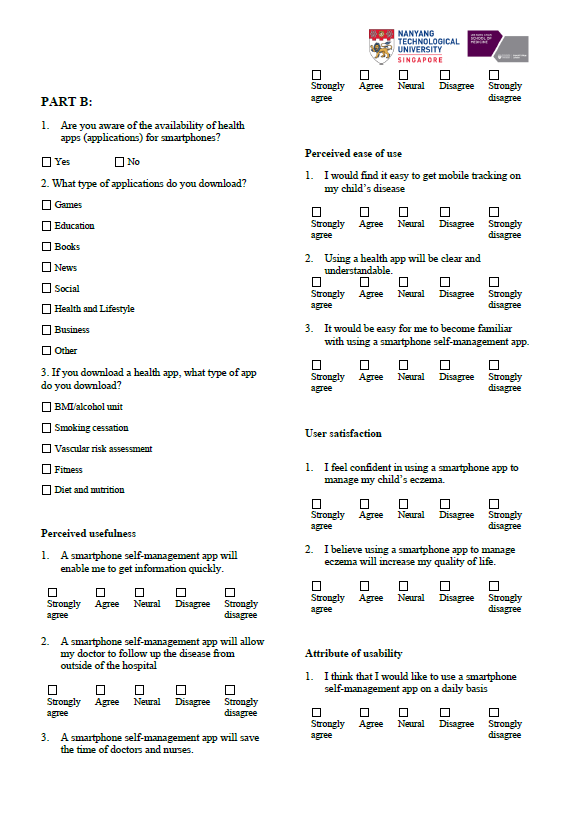

Supplement: Multimedia Appendix 1 [file mhealth_v8i10e16898_app1.docx]

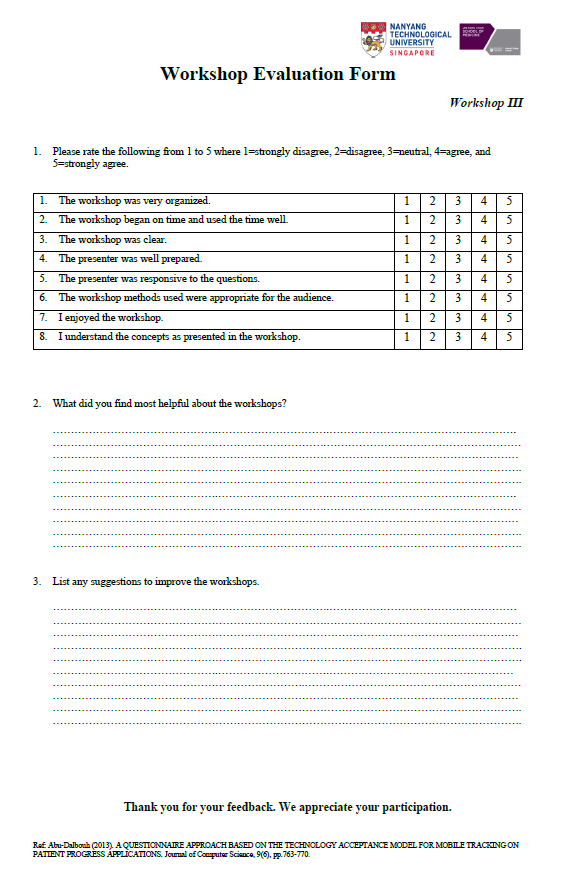
**Appendix 2.** Workshop evaluation form.

Supplement: Multimedia Appendix 2 [file mhealth_v8i10e16898_app2.docx]
