# Supplementary material for: Creating a Smartphone App for Caregivers of Children With Atopic Dermatitis With Caregivers, Health Care Professionals, and Digital Health Experts: Participatory Co-Design
Source: JMIR Mhealth Uhealth. 2020 Oct 29;8(10):e16898. doi: 10.2196/16898 (PMC7661237; doi:10.2196/16898)
Supplement: Multimedia Appendix 3 [file mhealth_v8i10e16898_app3.docx]

**Appendix 3. illustrative quotes.**

|  | Illustrative Quotes | |
| --- | --- | --- |
| Themes | **Caregivers** | **Healthcare professionals** |
| Empowerment by education | | |
| Understanding of causes, triggers, and symptoms of AD | [Quote 01] ‘We’re talking about temperature…sometimes it gets too cold [when] using the aircon…[which]…actually triggers the AD itself, so it will make the skin very, very dry and that’s when it gets terrible for him also. So, what kind of temperature is actually good for him? It's always a question, makes me wonder.’  (Caregiver 01, Female) |  |
|  | [Quote 02] ‘Even the doctor, he will diagnose this and tell you it’s a topical AD, but [when] this thing came, I didn't [know] whether it’s normal.’  (Caregiver 02, Female) |  |
| Understanding the disease course and the complications related to AD | [Quote 03] ‘Nobody tells you…I mean, the way it changes. I just so happened to come to this outing thing, and I saw, I mean, supposed to get AD trip outing and I saw a different one and I wasn’t given an education. It was later on [when I found out] he just had the same thing - just shed, shed, his skin just shed.’  (Caregiver 03, Female) | [Quote 05] ‘I think that information will come in, [i.e.] to recognize…the flare. In a way, if we could teach them [about the] cause [of and] how to identify the flare-up, that [would be] better. That is [the] key point of providing information. And most importantly, [teach them] to use their own language.’  (Doctor 01, Female) |
|  | [Quote 04] ‘When my son first had it, I know that he suddenly had lumps, so I wasn’t sure what was it. I brought him to see a doctor. At that time, he got blisters all over. I didn't know what it was. So, I found that link? It apparently, it’s linked with AD. I don't know why it’s linked but it’s linked. So, for me, that was very scary cause it’s just one of links, and stuff like that. So, for six months I didn't know what was happening - it was very frightening. If there is a way to let me know it beforehand, I will definitely be better prepared.’  (Caregiver 03, Female) | [Quote 06] ‘One thing that bothers me a lot is…the psychology of the parents. Because they don’t, they’re unable to accept that their kids have AD, that there is no cure, that there are many triggers. So…they try to find different and different ways to treat the AD. And no matter what your app is doing, [it has] to address…this fear or uncertainty that they have. Which is going to make it very difficult to, I don’t know, live [with] that treatment.’  (Doctor 03, Male) |
| Confusion over treatment |  |  |
| Which medication to choose and when to use it | [Quote 07] ‘What kind of salt [should] we use for them? What do they use to shower? When should I apply this cream? The shower creams. I cannot just buy any of the shower [creams]. We end up with having over ten tubes of cream at home, wish it was a little clear which one to use and when to use.’  (Caregiver 01, Female) |  |
|  | [Quote 08] ‘Different doctors’ prescriptions, different medications. That’s also one of the problems we face. My son is actually two years old. So, I brought him to the PD, initially, and he was prescribed with one cream which [contains] 1% steroid… Then when I brought him to another doctor, the doctor was like, “Your child is only a baby, two months old. How can you apply this cream on him? It's too strong for him.” So, another cream came, and that one came through this doctor and another cream came and we end up with all many, many, different steroid creams and totally no idea which one to use. Plus, different doctors have different knowledge…[and] tackle…problems differently.’  (Caregiver 01, Female) |  |
| Generic instruction vs specific instruction | [Quote 09] ‘I think about how to apply the cream and everything. Plus, when we go to the doctor, they don’t show us how to apply [it]. They just tell you, “This is the cream, you apply.” Any knowledge that is this sufficient? Am I [applying] too much, am I too little? How should I apply? Do I have to rub it? What? All [that information is] totally missing.’  (Caregiver 01, Female) |  |
| Alternative treatments preferable to steroids | [Quote 10] ‘I [keeping looking] for professional help; however, they always say that steroids [are] …the best treatments for AD. But I think there are other products as well, but they [insist on] staying on the steroids [and won’t talk about other] products. A lot of people [are choosing] organics, natural products and they think [they are] useful.’  (Caregiver 03, Female) | [Quote 11] ‘…’cause the community is very pushed back by steroids. You know, they say “it’s bad”, “it doesn’t work”, “everything else works”. We’re fighting [this] war.’  (Doctor 03, Female) |
| Emotional impact |  |  |
| Guilt and frustration | [Quote 12] ‘But you should say the guilt says that you never take care of your kids, probably. That’s why you have this thing. Because you never do this thing that causes problems. Where is the guilt initially? I had it.’  (Caregiver 08, Male) |  |
|  | [Quote 13] ‘I think people [keep] criticizing, [saying], “There must be something that you're doing during a pregnancy which resulted that your child having AD,” [or], “I probably shouldn't be eating this kind of stuff, shouldn't be doing this during pregnancy.” So, the whole process starts from the day you’re pregnant and you’re guilty until [the child is] born and then you’re guilty. Now if you do a C section, they say, “Yes, that’s also partly why your child has AD, plus, they didn’t go through the birth canal,” [or], “Ah, everything's your fault.”’  (Caregiver 01, Female) |  |
|  | [Quote 14] ‘People always tell me that don't put steroids on your son because it brings you the bad thing, so I always feel guilty of putting him steroid every single time. So, whenever I visit the doctor, I keep asking doctors about steroid, steroid, how bad it can be like that at the same time. So, being a mother, you want to try to listen to other people advices. The guilt, it's the guilt.’  (Caregiver 02, Female) |  |
|  | [Quote 15] ‘Because I will have people come [to] me, [saying]: “Why you give your son this kind of food? You should put him on a vegetable diet, a gluten-free diet.” As a parent initially, we go on a guilt trip, like, “Oh, we should have done this, maybe he would get better.” But initially, I'm sure it’s frustrating.’  (Caregiver 04, Female) |  |
| Self-esteem | [Quote 16] ‘My child [is] always very angry. I mean I’m sure it started with frustration because the skin conditions are on and off - but it shows as anger. It affects my child's self-esteem…harm his self-esteem.’  (Caregiver 08, Male) |  |
| The duty to make their child feel better | [Quote 17] ‘I think the main thing that I want [is] to make my child feel better, even though with myself I struggled a lot in the early stage. When my child [is] getting older and aware of herself…it starts to affect [her] emotional…health. My child currently is 2 years old, she doesn't feel anything yet, but on a longer-term she will start to get aware of herself and how others view her, and I think it will be another challenge that we need to meet as well. Not just the physical part, [but also] the physiological part.’  (Caregiver 02, Female) |  |
| Ideas and features of the AD self-management app |  |  |
|  |  | [Quote 18] ‘As they keep clicking that app, for example, [when] researching the triggers, after a while, the app…[should] push all the information related to triggers to the users, and eventually, it becomes interactive. The app will “know” the user.’  (Doctor 01, Male) |
